# The burden of hypertension, diabetes, and overweight/obesity by sedentary work pattern in Bangladesh: Analysis of Demographic and Health Survey 2017–18

**DOI:** 10.1371/journal.pgph.0002788

**Published:** 2024-02-06

**Authors:** Gulam Muhammed Al Kibria, Shakir Hossen, Dustin Gibson

**Affiliations:** 1 Department of International Health, Johns Hopkins University Bloomberg School of Public Health, Baltimore, Maryland, United States of America; 2 Department of Pulmonary, Critical Care and Sleep Medicine, University of Miami Miller School of Medicine, Miami, Florida, United States of America; Universiti Malaya, MALAYSIA

## Abstract

Most low- and middle-income countries, including Bangladesh, are currently undergoing epidemiologic and demographic transitions with an increasing burden of hypertension, diabetes, and overweight/obesity. Inadequate physical activity is a risk factor for these conditions and work-related activities contribute to most of the physical activities in Bangladesh. We investigated the association of the sedentary nature of occupation with hypertension, diabetes, and overweight/obesity in Bangladesh. If a person’s systolic/diastolic blood pressure, fasting plasma glucose concentration, and body mass index were ≥130/80 mmHg, ≥7 mmol/l, and ≥23 kg/m2, respectively, they were classified as hypertensive, diabetic, and overweight/obese. The nature of occupation/work was classified into three types: non-sedentary workers (NSW), sedentary workers (SW), and non-workers (NW). After describing the sample according to exposure and outcomes, we performed simple and multivariable logistic regression to investigate the association. Among 10900 participants (60.7% females, mean age: 40.0 years), about 43.2%, 13.2%, and 42.8% were NSW, SW, and NW, respectively. NSW, SW, NW, and overall people, respectively, had 6.7%, 14.5%, 11.7%, and 9.9% prevalence rates for diabetes; 18.0%, 32.9%, 28.3%, and 24.4% prevalence rates for overweight/obesity; and 18.0%, 32.9%, 38.3%, and 28.0% prevalence rate for hypertension. SW had higher odds of diabetes (AOR: 1.44, 95% CI: 1.15–1.81), overweight/obesity (AOR: 1.83, 95% CI: 1.52–2.21), and hypertension (AOR: 1.47, 95% CI: 1.21–1.77) than NSW. NW had higher odds of diabetes (AOR: 1.43, 95% CI: 1.19–1.71) or hypertension (AOR: 1.37, 95% CI: 1.22–1.56) but not higher odds of overweight/obesity (AOR: 1.11, 95% CI: 0.98–1.27) than NSW. We found higher prevalence and odds of the studied conditions among SW than NSW. Workplace physical activity programs may improve the physical activity and health of SW.

## Introduction

Globally, hypertension, diabetes, and overweight/obesity are among the leading causes of death and disabilities [1–3]. In recent decades, the burden of these conditions has become static in high-income countries; however, their burden continues to rise in many low- and middle-income countries (LMICs), including Bangladesh. LMICs have a high burden of communicable diseases as well; therefore, the increasing burden of non-communicable diseases (NCD) is posing an additional burden on the health system [4–6].

Recent nationally representative surveys, including Bangladesh Demographic and Health Surveys (BDHS) and STEPwise Approach to NCD Risk Factor Surveillance (STEPs), have estimated the prevalence and trends of NCDs [7–9]. These surveys also show that the prevalence of diabetes, overweight/obesity, and hypertension is rising in Bangladesh [7–9]. For example, the prevalence of hypertension increased from 20% to 34% in males and from 32% to 45% in females between BDHS conducted in 2011 and 2017 [7, 8].

Multiple studies also examined the factors associated with hypertension, diabetes, and overweight/obesity in Bangladesh and other similar LMICs [10–14]. These studies revealed that NCDs share some common risk factors, including older age, female gender, and socioeconomic status. Unlike high-income countries, the burden of these conditions is higher among people with higher socioeconomic status (i.e., higher education and/or wealth) [10, 13]. The association of sedentary lifestyles with NCDs like hypertension, diabetes, and overweight/obesity is well established [15, 16]. However, studying behavioral risk factors like sedentary lifestyles is challenging; it may be difficult to examine the association from cross-sectional data. For instance, a person may be more physically active after having the diagnosis of high blood pressure, blood sugar, or body weight; therefore, during the time of data collection, we may observe a higher prevalence of these conditions among them (i.e., reverse causality). The ‘sedentary work, job, profession, or occupation nature’ of a person could be less variable than the ‘sedentary behavioral lifestyle’. The STEPs 2018 reported that the vast majority of people met physical activity recommendations (about 90%); however, 73.4% of the total physical activity was contributed by work [17]. Therefore, a person may be more physically active due to the nature of the job and the nature of work could serve as a proxy measure for regular physical activity. Although studies from other countries have also reported the association of NCDs with the sedentary nature of work [18–20], little is known about this in Bangladesh.

This study was undertaken to examine the association of sedentary work nature with hypertension, diabetes, and overweight/obesity in Bangladesh. We also accounted for the non-workers (NW). We hypothesized that sedentary workers (SW) or NW would have a higher burden of these conditions than non-sedentary workers (NSW). Our findings will be helpful for policymakers and researchers to implement or design workplace programs promoting physical activity among workers.

## Methods

### Ethics statement

The institutional review boards of the ICF International and Bangladesh Medical Research Council provided ethical approval for the BDHS 2017–18.

### Data source

This cross-sectional study was conducted using BDHS 2017–18 data. As mentioned previously, BDHS 2017–18 was a nationally representative survey in Bangladesh. Mitra and Associates, a private research organization in Bangladesh, conducted the survey. Data collection took place between October 2017 and March 2018. We analyzed the data and prepared this report in November 2023 [7].

BDHS 2017–18 aimed to estimate the prevalence of hypertension and diabetes among men and women in Bangladesh. For measuring body mass index (BMI), data from men and ever-married women were collected. The survey included rural and urban regions of all administrative divisions of Bangladesh. It was the eighth DHS in the country. Briefly, a stratified, two-stage sample of households was chosen. A list of enumeration areas (EAs) from the 2011 Population and Housing Census of the People’s Republic of Bangladesh was first created for the sample frame. There were 425 EAs from rural and 250 from urban regions. The main sampling unit, EA, was chosen with a probability proportionate to its size. A comprehensive list of every household was completed in the EA. Then, from each EA, an average of 30 households were chosen for the second round. In this manner, 20,250 households in all were chosen. One-fourth of these homes were chosen at random to have their blood pressure and fasting plasma glucose levels measured. To measure blood pressure and fasting plasma glucose, all individuals residing in these houses (i.e., those who are 18 years of age or older) were invited to participate. Approximately 90% of the 14,704 eligible men and women took part. Four quality control teams from the data gathering organization monitored the quality of the data collection. The surveys underwent translation and validation within the Bangladeshi setting. The survey design, methods, sample size calculation, questionnaires, and other statistics are available online [7].

### Exposures

Participants reported their type of occupation. The dataset contains them as groups (S1 Table). From the list of occupations, we categorized the following groups as NSW (coded as 0): "farmer"; "agricultural worker"; "fisherman"; "poultry raising, cattle raising"; "home-based manufacturing (handicraft, food products)"; "rickshaw driver, brick breaking, road building, construction worker, boatman, and earthwork, etc."; "domestic servant"; and "non-agricultural worker (factory worker, blue-collar service)”.

The following groups were categorized as SW (coded as 1): "land owner"; "doctor, lawyer, dentist, accountant, teacher, nurse, family welfare visitor, mid and high-level services (government/private)"; "big businessman"; "small business/trader". People who were not "not working" and "retired" were considered NW (coded as 2).

### Outcomes

If a person met one of the three requirements—systolic blood pressure (SBP) of 140 mmHg (or more), diastolic blood pressure (DBP) of 90 mmHg (or more), or the participant stated that s/he was taking an antihypertensive medication, were considered hypertensive [21]. LIFE SOURCE UA-767 Plus monitors were used to record blood pressure. There were three measurements, spaced ten minutes apart. Additional data were recorded throughout those 10-minute intervals. The final blood pressure reading was reported using the mean of the second and third pressure measures [7]. Individuals who had a fasting plasma glucose level of greater than 7.0 mmol/l or were on antidiabetic medication to regulate their blood sugar levels were classified as diabetics [7]. The HemoCue 201+ blood analyzer was used to measure the blood sugar. Weight (in kilograms) divided by height (in meters squared) yielded the BMI. The average of the two digital weighing scale readings was used to determine the participants’ weight, while the standard clinical height scale was used to measure the participants’ height three times. We classified an individual as overweight or obese using the World Health Organization (WHO) specific suggested threshold (BMI 25 kg/m^2^) [22, 23].

### Other variables

We prepared a direct acyclic graph (i.e., conceptual framework) to investigate the association (S1 Fig). Potential confounding variables were selected based on published literature and data structure. These were age, gender, education, wealth status, place of residence, and division of residence. Participants reported their age as a continuous variable; it was categorized as 18–34, 35–44, 45–54, 55–64, and ≥65 years. Gender (i.e., male or female) was also self-reported. Urban residents were defined as those who were residing in a municipal or city corporation at the time of the survey. There were eight administrative divisions in Bangladesh at the time of the survey: Dhaka, Chattogram, Rajshahi, Khulna, Barisal, Sylhet, Rangpur, and Mymensingh. Education level was grouped as ’no formal education’, ‘primary (i.e., 1–5 school years)’, ‘secondary (i.e., 6–10 school years)’, and ‘college or above (i.e., 11 or more school years)’. In addition, participants were asked about the items in their homes, including household construction materials. Principal component analysis was utilized to derive the wealth index score, which was further categorized into quintiles (i.e., poorest, poorer, middle, richer, and richest) [7].

### Statistical analysis

First, we reported the overall sociodemographic characteristics according to occupation nature. Then, we compared the participants according to their presence of hypertension, diabetes, and overweight/obesity. We used mean and standard errors (SE) to report continuous variables (e.g., age) and tested them using analysis of variance or Student’s t-tests. For categorical variables (e.g., gender), we used weighted percentages (%) and unweighted numbers (n) to report them; and tested with chi-square tests. At last, we used simple and multivariable logistic regression to investigate the association of occupation nature and outcomes. Variables that were associated with exposure (i.e., occupation) and outcomes were included in the multivariable models. We reported both the unadjusted and adjusted odds ratios (OR) along with 95% confidence intervals (CIs). To report all the estimates, we took the dataset’s hierarchical structure (i.e., multistage cluster-sampling design) into consideration and applied the sample weights. The analyses were conducted with R (R Core Team, 2023).

## Results

A total of 7,932 participants (mean age: 39.2 (SE: 0.2), 60.7% females, and 25.5% urban residents) were included in the analysis (Table 1). The proportion of NSW, SW, and NW was 43.2% (n = 4650), 13.2% (n = 1517), and 42.8% (n = 4733), respectively. The proportion of older people or females was higher among NW than NSW or SW. SW had a higher proportion of college-educated people (36.5%) than NSW (4.7%) or NW (16.1%); similar distributions were observed for higher wealth quintiles or urban residence. The unweighted number of participants in each working group was reported in S1 Table.

**Table 1:** Comparison of the study sample based on occupation.

Variables		Overall (n = 10900)	Occupation type			
			Non-sedentary (n = 4650, 43.9%)	Sedentary (n = 1517, 13.2%)	Not working (n = 4733, 42.8%)	p-value
Age (in years)	Mean (SE)	40.0 (0.2)	40.4 (0.2)	40.0 (0.4)	39.7 (0.3)	0.12
	18 to 34	43.8 (4735)	38.4 (1781)	37.1 (555)	51.4 (2399)	<0.001
	35 to 44	19.9 (2172)	24.3 (1136)	27.9 (432)	13.0 (604)	
	45 to 54	14.1 (1564)	18.0 (842)	16.7 (259)	9.5 (463)	
	55 to 64	12.0 (1309)	12.8 (585)	12.0 (182)	11.2 (11.5)	
	65 or more	10.1 (1120)	6.6 (306)	6.3 (89)	14.9 (15.3)	
Gender	Female	60.7 (6609)	51.4 (2399)	15.3 (238)	84.3 (3972)	<0.001
	Male	39.3 (4291)	48.6 (2251)	84.7 (1279)	15.7 (761)	
Education level	No education	27.7 (2895)	33.3 (1502)	12.2 (178)	26.8 (1215)	<0.001
	Primary	29.7 (3283)	35.8 (1733)	22.7 (334)	25.7 (1216)	
	Secondary	28.7 (3046)	26.2 (1205)	28.6 (403)	31.4 (1438)	
	College or above	13.8 (1676)	4.7 (210)	36.5 (602)	16.1 (864)	
Wealth quintile	Poorest	19.9 (2184)	28.5 (1360)	8.6 (132)	14.5 (692)	<0.001
	Poorer	19.7 (2075)	25.3 (1166)	12.3 (180)	16.2 (729)	
	Middle	20.4 (2134)	20.4 (963)	18.6 (254)	20.9 (917)	
	Richer	19.8 (2112)	17.3 (785)	22.7 (327)	21.6 (1000)	
	Richest	20.3 (2395)	8.5 (376)	37.9 (624)	26.8 (1395)	
Place of residence	Urban	25.5 (3765)	18.6 (1179)	38.3 (757)	28.7 (1829)	0.19
	Rural	74.5 (7135)	81.4 (3471)	61.7 (760)	71.3 (2904)	
Division of residence	Dhaka	23.4 (1439)	19.2 (510)	29.9 (251)	25.6 (678)	<0.001
	Chattagram	16.9 (1453)	11.4 (453)	16.1 (171)	22.7 (536)	
	Barishal	5.7 (1160)	5.4 (433)	5.6 (196)	5.9 (824)	
	Khulna	12.4 (1486)	13.5 (661)	11.7 (210)	11.3 (615)	
	Mymensingh	8.2 (1235)	9.7 (619)	6.9 (152)	6.9 (464)	
	Rajshahi	14.2 (1400)	17.4 (714)	12.5 (184)	11.3 (502)	
	Rangpur	13.0 (1455)	18.2 (839)	11.6 (189)	8.1 (427)	
	Sylhet	6.5 (1272)	5.1 (421)	5.8 (164)	8.2 (687)	
Diabetes	Mean FPG (SE) mmol/L	5.7 (0.02)	5.5 (0.03)	6.0 (0.05)	5.8 (0.03)	<0.001
	No	90.1 (9832)	93.2 (4343)	85.5 (1298)	88.3 (4191)	<0.001
	Yes	9.9 (1068)	6.7 (307)	14.5 (219)	11.7 (542)	
Over-weight/Obesity	Mean BMI (SE) kg/m^2^	22.4 (0.1)	21.7 (0.1)	23.3 (0.1)	22.8 (0.1)	<0.001
	No	75.6 (8213)	82.0 (3799)	67.1 (1037)	71.7 (3377)	<0.001
	Yes	24.4 (2687)	18.0 (851)	32.9 (480)	28.3 (1356)	
Hyper-tension	Mean SBP (SE) mmHg	122.6 (0.3)	121.3 (0.4)	125.8 (0.8)	122.9 (0.4)	<0.001
	Mean DBP (SE) mmHg	80.3 (0.2)	79.9 (0.2)	82.7 (0.4)	80.2 (0.2)	<0.001
	No	71.9 (7771)	82.0 (3799)	67.1 (1037)	71.7 (3377)	<0.001
	Yes	28.0 (3129)	18.0 (851)	32.9 (480)	28.3 (1356)	

Abbreviations: BMI: Body mass index, DBP: Diastolic blood pressure, FPG: Fasting plasma glucose; SBP: Systolic blood pressure, SE: Standard error

**Fig 1:**
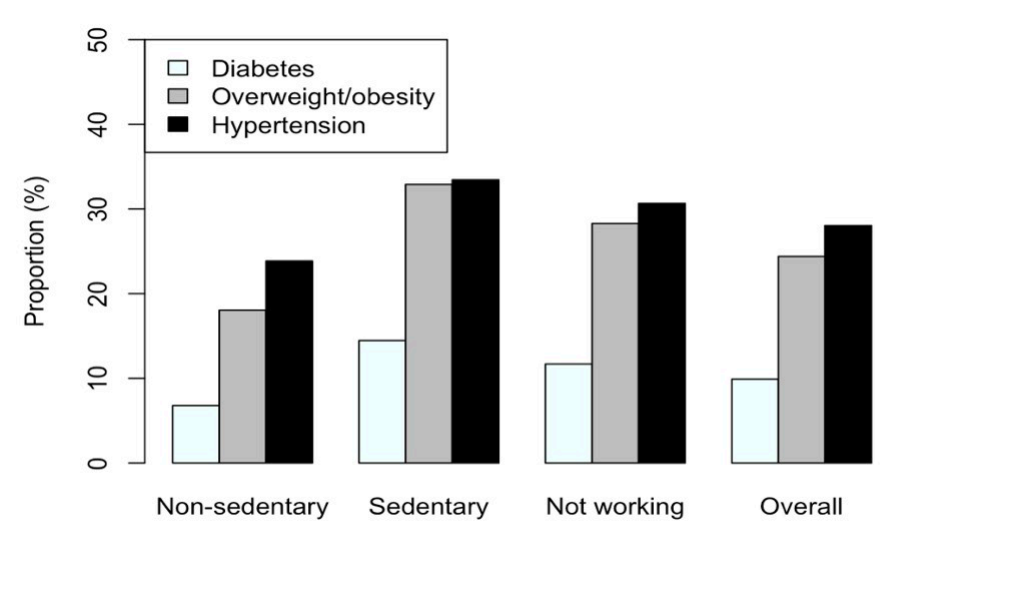
Prevalence of diabetes, overweight/obesity, and hypertension by occupation.

Fig 1 shows the prevalence of three studied conditions according to occupational nature, along with the overall prevalence. NSW, SW, NW, and overall sample, respectively, had 6.7%, 14.5%, 11.7%, and 9.9% prevalence rates for diabetes; 18.0%, 32.9%, 28.3%, and 24.4% prevalence rates for overweight/obesity; and 18.0%, 32.9%, 38.3%, and 28.0% prevalence rates for hypertension.

When we compared the sample characteristics of diabetic, overweight/obese, and hypertensive people with the overall sample (Table 2), diabetic (46.7 years (SE: 0.6)) and hypertensive (49.3 years (SE: 0.3)) people had a relatively higher mean age than overall people (40.0 years (SE: 0.2)) or overweight/obese (39.4 years (SE: 0.3)). Overweight/obese people had a higher proportion of females than other groups or the overall sample. People with diabetes (37.3%), overweight/obesity (37.9%), and hypertension (24.1%) had a higher proportion of people with the richest wealth quintile than the overall sample. S2–S4 Tables show the comparison of sample based on diabetes, overweight/obesity, and hypertension.

**Table 2:** Comparison of the sample based on diabetes, overweight/obesity, and hypertension.

Variables		Overall (n = 10900)	Presence of condition		
			Diabetes (n = 1068)	Overweight/Obesity (n = 2687)	Hypertension (n = 3129)
Age (in years)	Mean (SE)	40.0 (0.2)	46.7 (0.6)	39.4 (0.3)	49.3 (0.3)
	18 to 34	43.8 (4735)	23.2 (246)	41.7 (1108)	19.7 (600)
	35 to 44	19.9 (2172)	23.2 (245)	26.1 (691)	20.5 (637)
	45 to 54	14.1 (1564)	20.3 (215)	16.6 (446)	19.9 (623)
	55 to 64	12.0 (1309)	18.4 (200)	10.0 (278)	19.9 (630)
	65 or more	10.1 (1120)	14.8 (162)	5.7 (164)	20.0 (639)
Gender	Female	60.7 (6609)	58.5 (644)	71.8 (1938)	61.9 (1940)
	Male	39.3 (4291)	41.5 (424)	28.2 (749)	38.1 (1189)
Education level	No education	27.7 (2895)	27.4 (275)	18.2 (456)	35.5 (1048)
	Primary	29.7 (3283)	30.2 (325)	28.1 (745)	945 (29.4)
	Secondary	28.7 (3046)	29.6 (305)	35.4 (920)	24.3 (738)
	College or above	13.8 (1676)	12.8 (163)	18.3 (566)	10.9 (398)
Wealth quintile	Poorest	19.9 (2184)	11.0 (119)	9.1 (243)	17.2 (519)
	Poorer	19.7 (2075)	12.1 (125)	13.0 (326)	17.9 (531)
	Middle	20.4 (2134)	16.7 (172)	17.4 (451)	20.3 (610)
	Richer	19.8 (2112)	23.0 (234)	22.5 (563)	20.5 (639)
	Richest	20.3 (2395)	37.3 (418)	37.9 (1104)	24.1 (830)
Place of residence	Urban	25.5 (3765)	34.3 (463)	35.0 (1225)	26.5 (1134)
	Rural	74.5 (7135)	65.7 (605)	65.0 (1462)	73.5 (1995)
Division of residence	Dhaka	23.4 (1439)	33.4 (213)	28.4 (445)	20.1 (353)
	Chattagram	16.9 (1453)	18.8 (162)	20.6 (441)	18.5 (449)
	Barishal	5.7 (1160)	5.7 (122)	5.3 (288)	11.9 (378)
	Khulna	12.4 (1486)	10.5 (137)	12.9 (409)	13.4 (463)
	Mymensingh	8.2 (1235)	6.8 (104)	5.6 (222)	6.7 (290)
	Rajshahi	14.2 (1400)	11.2 (118)	12.4 (325)	14.3 (402)
	Rangpur	13.0 (1455)	7.4 (89)	9.9 (304)	14.2 (457)
	Sylhet	6.5 (1272)	6.2 (123)	4.9 (253)	6.1 (337)
Diabetes	No	90.1 (9832)		84.5 (2260)	83.7 (2618)
	Yes	9.9 (1068)		15.5 (427)	16.3 (511)
Overweight/Obesity	No	75.6 (8213)	61.8 (641)		63.3 (1989)
	Yes	24.4 (2687)	38.2 (427)		36.7 (1140)
Hypertension	No	75.6 (8213)	54.0 (557)	57.8 (1547)	
	Yes	24.4 (2687)	46.0 (511)	42.2 (1140)	

Abbreviations: BMI: Body mass index, SE: Standard error

As we can see in Table 3, SW had higher odds of diabetes (AOR: 1.44, 95% CI: 1.15–1.81), overweight/obesity (AOR: 1.83, 95% CI: 1.52–2.21), and hypertension (AOR: 1.47, 95% CI: 1.21–1.77) than NSW. Although NW had higher odds of diabetes (AOR: 1.43, 95% CI: 1.19–1.71) or hypertension (AOR: 1.37, 95% CI: 1.22–1.56) than NSW, they did not have higher odds of overweight/obesity (AOR: 1.11, 95% CI: 0.98–1.27).

**Table 3:** Associations of hypertension, diabetes, and overweight/obesity by occupation.

		Diabetes^1^	Overweight/Obesity^2^	Hypertension^3^
UOR (95% CI)	Sedentary	2.32***(1.87, 2.87)	2.22***(1.89, 2.62)	1.60***(1.36,1.89)
(Ref: Non-sedentary)	Not working	1.82***(1.55, 2.13)	1.79***(1.59, 2.01)	1.41***(1.27,1.57)
AOR (95% CI)	Sedentary	1.44**(1.15, 1.81)	1.83***(1.52, 2.21)	1.47***(1.21,1.77)
(Ref: Non-sedentary)	Not working	1.43***(1.19, 1.71)	1.11(0.98, 1.27)	1.37**(1.22,1.56)

Abbreviations: AOR: Adjusted odds ratio, CI: Confidence interval, UOR: Unadjusted odds ratio.

1. Adjusted for age, wealth quintile, division, overweight/obesity and hypertension

2. Adjusted for age, gender, wealth quintile, region

3. Adjusted for age, education, wealth quintile, division, diabetes, and overweight/obesity.

## Discussion

We observed that SW had a significantly higher prevalence and odds of hypertension, diabetes, and overweight/obesity than NSW. This finding supports our hypothesis regarding the association of the nature of occupation with these conditions. Although we found higher prevalence and odds of hypertension and diabetes among NW than NSW, they did not have higher odds of overweight/obesity. To our knowledge, this is the first epidemiological study to report the association of the nature of occupation with hypertension, diabetes, and overweight/obesity in Bangladesh.

It is well known that the three conditions we studied are related and share many common genetic and lifestyle risk factors, including physical activity [13, 24–27]. While genetic factors are not modifiable, the focus has to be stronger to improve lifestyle-related factors like physical activity. Physical activity reduces the level of blood pressure, blood sugar, or body weight. Furthermore, physical activity improves dyslipidemia, vascular function, hemostatic factors, mental health, brain function, physical function, sleep, and overall quality of life [24, 28, 29]. Physical activity would not only benefit people who want to prevent hypertension, diabetes, or overweight/obesity, but it would also help to control those people who have these conditions [24]. Overall, increased physical activity would have synergistic effects on physical and mental health, including on the overall health systems of the country.

As we mentioned earlier, our study aimed to investigate the type/nature of occupation as a measure of physical activity, since longitudinal data on physical activity was not available. Moreover, a majority of the physical activity in Bangladesh is achieved through activities related to work, and only a small proportion (5%) is achieved through recreational activities [17]. For adults, the WHO currently recommends having weekly physical activity of moderate intensity for 150–300 minutes or vigorous intensity for 75–150 minutes [30]. Therefore, improving physical activity is essential for SW as they have a higher burden of hypertension, diabetes, and overweight/obesity. It is important to provide workplace support to improve the physical activity of employees, especially those who are engaged in more sedentary occupations. Furthermore, Bangladesh and other similar nations may benefit from instituting national awareness and control programs to increase awareness and encourage employees to engage in more physical activity. Previous investigations showed that there was insufficient oversight and monitoring of previously put into place measures to identify and manage the population of people at risk [31]. Although previous studies reported the determinants of inadequate physical activity in Bangladesh [32, 33], future studies should also explore the barriers to sufficient physical activity for SW people. We also observed that about 43% of our study population were not working, and they had a higher burden of hypertension or diabetes as well, it is also essential to consider them during formulating a program, policy, or intervention. Future studies should also examine the impact of actual physical activity level each individual perform as a part of regular work, recreation, and travel. The physical activity guideline needs to provide guidance to individuals who perform more sedentary work and lifestyle.

Our study includes many noteworthy strengths. The survey encompassed both rural and urban areas across all administrative divisions, making the results broadly representative and applicable to residents across the nation. Second, BDHS recorded blood pressure and other metrics using defined and validated procedures, which improved the validity of our results. Both the response rate and sample size were high.

This study has some limitations as well. Because the datasets were cross-sectional, the conclusions might not be causal. Since the measurements for this survey were only collected over one day, the results do not reflect clinical diagnoses of diabetes or hypertension. We did not account for food habits or several other variables since there was insufficient data. The accuracy of the results could be impacted by the effectiveness or proficiency of the survey personnel who recorded the measurements [34]. These limitations, however, highlight the need for higher-quality statistics on them in Bangladesh.

### Conclusions

This study showed the association of occupation type/nature with three chronic conditions (i.e., hypertension, diabetes, and overweight/obesity) in Bangladesh. The higher burden of studied conditions among SW highlights the importance of workplace support to encourage more recreational physical activities among them. It is also essential to implement national awareness, prevention, and control programs to improve the overall levels of physical activity and to reduce the future burden of hypertension, diabetes, and overweight/obesity in the country.

## Supporting information

S1 Fig Conceptual framework. (TIFF)

S1 Table Number of participants from different occupational patterns. (DOCX)

S2 Table Comparison of the sample based on the presence of diabetes. (DOCX)

S3 Table Comparison of the sample based on the presence of overweight/obesity. (DOCX)

S4 Table Comparison of the sample based on the presence of hypertension. (DOCX)
