# Supplementary material for: The burden of hypertension, diabetes, and overweight/obesity by sedentary work pattern in Bangladesh: Analysis of Demographic and Health Survey 2017–18
Source: PLOS Glob Public Health. 2024 Feb 6;4(2):e0002788. doi: 10.1371/journal.pgph.0002788 (PMC10846693; doi:10.1371/journal.pgph.0002788)
Supplement: S1 Table — (DOCX) [file pgph.0002788.s002.docx]

S1 Table: Number of participants from different occupational patterns

| **Occupation groups** | N (%) |
| --- | --- |
| **Non-sedentary workers** |  |
| "Farmer" | 923 (8.5) |
| "Agricultural Worker" | 499 (4.6) |
| "Fisherman" | 54 (0.5) |
| "Poultry raising, Cattle raising" | 1826 (16.8) |
| "Home-based Manufacturing (Handicraft, Food products)" | 124 (1.1) |
| "Rickshaw driver, Brick breaking, Road building, Construction worker, Boatman, and Earth work etc." | 420 (3.9) |
| "Domestic servant" | 133 (1.2) |
| "Non-agricultural worker (Factory worker, blue collar service)" | 671 (6.2) |
| **Sedentary workers** |  |
| "Land Owner" | 13 (0.1) |
| "Doctor, Lawyer, Dentist, Accountant, Teacher, Nurse, Family welfare visitor, Mid and high level services (Government/private)" | 361 (3.3) |
| "Big businessman" | 99 (0.9) |
| "Small business/trader" | 1044 (9.6) |
| **Not working** |  |
| “Not working” | 4730 (43.4) |
| "Retired" | 2 (0.0) |
