# Supplementary material for: The burden of hypertension, diabetes, and overweight/obesity by sedentary work pattern in Bangladesh: Analysis of Demographic and Health Survey 2017–18
Source: PLOS Glob Public Health. 2024 Feb 6;4(2):e0002788. doi: 10.1371/journal.pgph.0002788 (PMC10846693; doi:10.1371/journal.pgph.0002788)
Supplement: S2 Table — (DOCX) [file pgph.0002788.s003.docx]

S2 Table: Comparison of the sample based on the presence of diabetes

| Variables | | Overall  (n = 10900) | Presence of diabetes | | |
| --- | --- | --- | --- | --- | --- |
|  |  |  | No  (n = 9832) | Yes (n=1068) | p-values |
| Age (in years) | 18 to 34 | 43.8 (4735) | 46.1 (4489) | 23.2 (246) | <0.001 |
|  | 35 to 44 | 19.9 (2172) | 19.6 (1927) | 23.2 (245) |  |
|  | 45 to 54 | 14.1 (1564) | 13.5 (1349) | 20.3 (215) |  |
|  | 55 to 64 | 12.0 (1309) | 11.3 (1109) | 18.4 (200) |  |
|  | 65 or more | 10.1 (1120) | 9.6 (958) | 14.8 (162) |  |
| Gender | Female | 60.7 (6609) | 60.9 (5965) | 58.5 (644) | 0.14 |
|  | Male | 39.3 (4291) | 39.1 (3867) | 41.5 (424) |  |
| Education level | No education | 27.7 (2895) | 27.7 (2620) | 27.4 (275) | 0.78 |
|  | Primary | 29.7 (3283) | 29.7 (2958) | 30.2 (325) |  |
|  | Secondary | 28.7 (3046) | 28.6 (2741) | 29.6 (305) |  |
|  | College or above | 13.8 (1676) | 13.9 (1513) | 12.8 (163) |  |
| Wealth quintile | Poorest | 19.9 (2184) | 20.8 (2065) | 11.0 (119) | <0.001 |
|  | Poorer | 19.7 (2075) | 20.5 (1950) | 12.1 (125) |  |
|  | Middle | 20.4 (2134) | 20.8 (1962) | 16.7 (172) |  |
|  | Richer | 19.8 (2112) | 19.5 (1878) | 23.0 (234) |  |
|  | Richest | 20.3 (2395) | 18.4 (1977) | 37.3 (418) |  |
| Place of residence | Urban | 25.5 (3765) | 24.6 (3302) | 34.3 (463) | <0.001 |
|  | Rural | 74.5 (7135) | 75.4 (6530) | 65.7 (605) |  |
| Division of residence | Dhaka | 23.4 (1439) | 22.3 (1226) | 33.4 (213) | <0.001 |
|  | Chattagram | 16.9 (1453) | 16.7 (1291) | 18.8 (162) |  |
|  | Barishal | 5.7 (1160) | 5.7 (1038) | 5.7 (122) |  |
|  | Khulna | 12.4 (1486) | 12.5 (1349) | 10.5 (137) |  |
|  | Mymensingh | 8.2 (1235) | 8.3 (1131) | 6.8 (104) |  |
|  | Rajshahi | 14.2 (1400) | 14.5 (1282) | 11.2 (118) |  |
|  | Rangpur | 13.0 (1455) | 13.6 (1366) | 7.4 (89) |  |
|  | Sylhet | 6.5 (1272) | 6.5 (1149) | 6.2 (123) |  |
| Overweight/  Obesity | No | 75.6 (8213) | 77.1 (7572) | 61.8 (641) | <0.001 |
|  | Yes | 24.4 (2687) | 22.9 (2260) | 38.2 (427) |  |
| Hypertension | No | 75.6 (8213) | 73.9 (7214) | 54.0 (557) | <0.001 |
|  | Yes | 24.4 (2687) | 26.1 (2618) | 46.0 (511) |  |
