# Supplementary material for: The burden of hypertension, diabetes, and overweight/obesity by sedentary work pattern in Bangladesh: Analysis of Demographic and Health Survey 2017–18
Source: PLOS Glob Public Health. 2024 Feb 6;4(2):e0002788. doi: 10.1371/journal.pgph.0002788 (PMC10846693; doi:10.1371/journal.pgph.0002788)
Supplement: S3 Table — (DOCX) [file pgph.0002788.s004.docx]

S3 Table: Comparison of the sample based on the presence of overweight/obesity

| Variables | | Overall  (n = 10900) | Presence of Overweight/  Obesity | | P-values |
| --- | --- | --- | --- | --- | --- |
|  |  |  | No (n = 8213) | Yes (n=2687) |  |
| Age (in years) | 18 to 34 | 43.8 (4735) | 44.5 (3627) | 41.7 (1108) | <0.001 |
|  | 35 to 44 | 19.9 (2172) | 18.0 (1481) | 26.1 (691) |  |
|  | 45 to 54 | 14.1 (1564) | 13.4 (1118) | 16.6 (446) |  |
|  | 55 to 64 | 12.0 (1309) | 12.6 (1031) | 10.0 (278) |  |
|  | 65 or more | 10.1 (1120) | 11.5 (956) | 5.7 (164) |  |
| Gender | Female | 60.7 (6609) | 57.1 (4671) | 71.8 (1938) | <0.001 |
|  | Male | 39.3 (4291) | 42.9 (3542) | 28.2 (749) |  |
| Education level | No education | 27.7 (2895) | 30.7 (2439) | 18.2 (456) | <0.001 |
|  | Primary | 29.7 (3283) | 30.3 (2538) | 28.1 (745) |  |
|  | Secondary | 28.7 (3046) | 26.6 (2126) | 35.4 (920) |  |
|  | College or above | 13.8 (1676) | 12.4 (1110) | 18.3 (566) |  |
| Wealth quintile | Poorest | 19.9 (2184) | 23.3 (1941) | 9.1 (243) | <0.001 |
|  | Poorer | 19.7 (2075) | 21.8 (1749) | 13.0 (326) |  |
|  | Middle | 20.4 (2134) | 21.3 (1683) | 17.4 (451) |  |
|  | Richer | 19.8 (2112) | 19.0 (1549) | 22.5 (563) |  |
|  | Richest | 20.3 (2395) | 14.6 (1291) | 37.9 (1104) |  |
| Place of residence | Urban | 25.5 (3765) | 22.5 (2540) | 35.0 (1225) | <0.001 |
|  | Rural | 74.5 (7135) | 77.5 (5673) | 65.0 (1462) |  |
| Division of residence | Dhaka | 23.4 (1439) | 21.7 (994) | 28.4 (445) | <0.001 |
|  | Chattagram | 16.9 (1453) | 15.7 (1012) | 20.6 (441) |  |
|  | Barishal | 5.7 (1160) | 5.8 (872) | 5.3 (288) |  |
|  | Khulna | 12.4 (1486) | 12.1 (1077) | 12.9 (409) |  |
|  | Mymensingh | 8.2 (1235) | 9.0 (1013) | 5.6 (222) |  |
|  | Rajshahi | 14.2 (1400) | 14.7 (1075) | 12.4 (325) |  |
|  | Rangpur | 13.0 (1455) | 14.0 (1151) | 9.9 (304) |  |
|  | Sylhet | 6.5 (1272) | 7.0 (1019) | 4.9 (253) |  |
| Diabetes | No | 90.1 (9832) | 91.9 (7572) | 84.5 (2260) | <0.001 |
|  | Yes | 9.9 (1068) | 8.1 (641) | 15.5 (427) |  |
| Hypertension | No | 75.6 (8213) | 76.5 (6224) | 57.8 (1547) | <0.001 |
|  | Yes | 24.4 (2687) | 23.5 (1989) | 42.2 (1140) |  |
