# Supplementary material for: The burden of hypertension, diabetes, and overweight/obesity by sedentary work pattern in Bangladesh: Analysis of Demographic and Health Survey 2017–18
Source: PLOS Glob Public Health. 2024 Feb 6;4(2):e0002788. doi: 10.1371/journal.pgph.0002788 (PMC10846693; doi:10.1371/journal.pgph.0002788)
Supplement: S4 Table — (DOCX) [file pgph.0002788.s005.docx]

S4 Table: Comparison of the sample based on the presence of hypertension

| Variables | | Overall  (n = 10900) | Presence of hypertension | | P-values |
| --- | --- | --- | --- | --- | --- |
|  |  |  | No (n = 7771) | Yes (n=3129) |  |
| Age (in years) | 18 to 34 | 43.8 (4735) | 53.2 (4135) | 19.7 (600) | <0.001 |
|  | 35 to 44 | 19.9 (2172) | 19.7 (1535) | 20.5 (637) |  |
|  | 45 to 54 | 14.1 (1564) | 12.0 (941) | 19.9 (623) |  |
|  | 55 to 64 | 12.0 (1309) | 8.9 (679) | 19.9 (630) |  |
|  | 65 or more | 10.1 (1120) | 6.3 (481) | 20.0 (639) |  |
| Gender | Female | 60.7 (6609) | 60.2 (4669) | 61.9 (1940) | 0.12 |
|  | Male | 39.3 (4291) | 39.8 (3102) | 38.1 (1189) |  |
| Education level | No education | 27.7 (2895) | 24.7 (1847) | 35.5 (1048) | <0.001 |
|  | Primary | 29.7 (3283) | 29.9 (2338) | 945 (29.4) |  |
|  | Secondary | 28.7 (3046) | 30.5 (2308) | 24.3 (738) |  |
|  | College or above | 13.8 (1676) | 15.0 (1278) | 10.9 (398) |  |
| Wealth quintile | Poorest | 19.9 (2184) | 20.9 (1665) | 17.2 (519) | <0.001 |
|  | Poorer | 19.7 (2075) | 20.4 (1544) | 17.9 (531) |  |
|  | Middle | 20.4 (2134) | 20.4 (1524) | 20.3 (610) |  |
|  | Richer | 19.8 (2112) | 19.5 (1473) | 20.5 (639) |  |
|  | Richest | 20.3 (2395) | 18.8 (1565) | 24.1 (830) |  |
| Place of residence | Urban | 25.5 (3765) | 25.2 (2631) | 26.5 (1134) | 0.24 |
|  | Rural | 74.5 (7135) | 74.8 (5140) | 73.5 (1995) |  |
| Division of residence | Dhaka | 23.4 (1439) | 24.6 (1086) | 20.1 (353) | <0.001 |
|  | Chattagram | 16.9 (1453) | 16.3 (1004) | 18.5 (449) |  |
|  | Barishal | 5.7 (1160) | 5.3 (782) | 11.9 (378) |  |
|  | Khulna | 12.4 (1486) | 11.9 (1023) | 13.4 (463) |  |
|  | Mymensingh | 8.2 (1235) | 8.7 (945) | 6.7 (290) |  |
|  | Rajshahi | 14.2 (1400) | 14.1 (998) | 14.3 (402) |  |
|  | Rangpur | 13.0 (1455) | 12.5 (998) | 14.2 (457) |  |
|  | Sylhet | 6.5 (1272) | 6.7 (935) | 6.1 (337) |  |
| Diabetes | No | 90.1 (9832) | 92.6 (7214) | 83.7 (2618) | <0.001 |
|  | Yes | 9.9 (1068) | 7.4 (557) | 16.3 (511) |  |
| Overweight/  Obesity | No | 75.6 (8213) | 80.4 (6224) | 63.3 (1989) | <0.001 |
|  | Yes | 24.4 (2687) | 19.6 (1547) | 36.7 (1140) |  |
